# The charitable food system as a change agent

**DOI:** 10.3389/fpubh.2023.1156501

**Published:** 2023-03-31

**Authors:** Marlene B. Schwartz, Caitlin E. Caspi

**Affiliations:** ^1^Rudd Center for Food Policy and Health, University of Connecticut, Hartford, CT, United States; ^2^Department of Human Development and Family Sciences, University of Connecticut, Storrs, CT, United States; ^3^Department of Allied Health Sciences, University of Connecticut, Storrs, CT, United States

**Keywords:** food insecurity, charitable food, nutrition, food bank, food pantry

## 1. Introduction

In 2021, 10.2% of United States (U.S.) households reported food insecurity as measured by the United States Department of Agriculture (USDA) annual survey (1). The US government spent $182.5 billion in fiscal year 2021 on federal food programs, a significant increase from the previous year (1). Despite this investment, there are individuals who do not qualify for federal assistance or need additional help. In 2021 an estimated 53 million people turned to a network of food banks and food pantries known as the “charitable food system” to obtain food at no cost (2).

The U.S. charitable food system has the potential to meaningfully address social determinants of health. First, this network has an expansive reach. In the U.S., many charitable food agencies are under the umbrella of Feeding America, a national network of ~200 food banks and 45,000 food pantries that reach communities throughout the country (3). By way of comparison, there are ~27,000 high schools in the U.S. (4). Second, the sites where food is distributed are deeply connected to specific communities. Food pantries are typically located in faith-based or community organizations and serve defined geographic areas. Because they are hyperlocal, pantries can provide customized and culturally tailored services to meet the needs of nearby residents.

Some experts criticize charitable food by calling it a short-term solution that does not help change the systems that led to the problem of food insecurity, and worse, allows the government to shirk its responsibility to ensure that people are fed (5). While we do not suggest that the charitable food system can “go it alone” in addressing food insecurity, in this paper, we do suggest ways that the public health community can tap the system's potential to strengthen community health and voices. We highlight (a) strategies to prioritize access to nutritious food and provide a dignified experience; (b) examples of how food pantries can be a portal to federal benefits, health care, and other resources; and (c) ideas on how these non-profit agencies can increase civic engagement and raise community voices.

## 2. Maximizing nutrition in food banks

Historically, food banks and pantries measured success by the pounds of food they distributed. As a result, the quantity of food was prioritized over quality, and the nutritional value of the foods available was limited (6–8). About a decade ago, a national study found that people who use the charitable food system are at greater risk of diet-related chronic diseases than other populations (3). Specifically, 33.2% reported that they had someone in the household with diabetes, and 57.8% reported a household member with high blood pressure (3). At that time, some food bankers had implemented policies and practices to improve nutrition, but others were concerned that a focus on nutrition would harm donor relationships (9).

Support for nutrition policies grew as research documented that clients clearly expressed that they want healthy options when they visit food pantries. For example, a statewide survey of Minnesota food pantry clients found that the most requested items were fresh fruits and vegetables, dairy, and other staples such as meat, eggs, and cooking items (10). Clients also clearly expressed that they preferred these items over common and ubiquitous offerings, such as non-perishable canned soups and boxed meals (10). Additional studies measuring client demand across the U.S. show remarkable consistency in these preferences, particularly in the demand for fresh fruits and vegetables and dairy (11–15). These data challenged long-held notions that clients don't want healthy food or don't know how to prepare healthy food (16–19).

Although identifying nutritious foods might seem like a straightforward task, the nuances and complexity of nutrition were exhibited in the range of ranking and scoring systems developed across segments of the charitable food system (20–25). The risks of having multiple systems in place are inconsistent nutrition messaging, and difficulty tracking national progress on improving the nutritional quality of food bank inventory. To address this, a panel of researchers and charitable food agency leaders was convened in 2019 for a year-long project to develop the Healthy Eating Research (HER) Nutrition Guidelines for the Charitable Food System (26). These guidelines, released in 2020, offer a process for ranking most food items into three categories: green=choose often, yellow=choose sometimes, or red=choose rarely based on levels of saturated fat, sodium, and added sugar (26). Feeding America has adopted and promoted these guidelines in its food bank network (27). The guidelines have also been adapted to create messages for retailers and distributors to improve the nutritional quality of corporate donations (28). These recent developments represent the first time that the U.S. has had a nationally accepted set of nutrition standards and an implementation process to guide the charitable food system in improving its food supply.

## 3. Improving the food pantry experience

Just as food banks were assessed by pounds distributed, food pantry success was further measured by the number of people equitably served in a specific window of time. As a result, efficiency and fairness were prioritized and a common strategy was to compile the same assortment of foods in pre-packed bags (sometimes adjusted by family size) and hand them out to clients who stood in line. Although this guarantees equal treatment, this strategy denies clients the opportunity to choose their preferred foods or refuse foods they do not want or can't eat. Further, it reinforces that this is a “hand-out.” Because of these concerns, the network has shifted significantly to “client choice” pantries where people can choose their own foods. Although there are still often limits to the number of specific foods that can be taken, the experience more closely resembles shopping in a small store.

The combined shifts toward client choice and healthier foods have inspired efforts nationally to redesign pantries to create a more dignified and less stigmatizing experience for clients (29). Recent interventions to the food pantry environment have included technical assistance to food pantry staff and volunteers to provide healthier food (30–32) and environmental “nudges” to promote behavior change among clients (33). The latter includes behavioral economics strategies, which aim to change behavior through careful choice architecture. In the food pantry, this might include changing defaults (e.g., removing boxed meals as a default selection); changing the order in which people encounter food (e.g., offering the healthiest food first); and offering other cues for the selection of healthy food (e.g., signage, bundling, and recipes) (34–36).

A growing number of studies show that nudges are effective at promoting healthier choices in the charitable food system (37–41). The Supporting Wellness at Pantries (SWAP) toolkit (42) shows how to incorporate awareness of the HER nutrition standards as foods travel from the donor to the client. When this is done, there is evidence that staff, volunteers, and clients shift their behavior and choose healthier foods (30, 41, 43). The pantry setting also provides a unique opportunity to encourage clients to try new, healthier products. Research suggests that shoppers who are low-income may be reluctant to risk buying an unfamiliar product that their family may not like or eat (44). Food pantries allow people to take home unfamiliar foods without the same level of financial risk.

Another community-led intervention undergoing rigorous evaluation, SuperShelf, offered technical assistance to food pantries to transform their physical space and maximize choices for clients (45). The physical transformations included colorful paint, new produce displays, and reorganization into food groups. Healthy food was arranged so that it looked abundant and appetizing, as it does when shopping in a grocery store (46). Shopping lists were redesigned to offer both more flexibility and more healthy choices to clients. Staff and volunteers noted that the physical transformation was a conduit to other changes, such as improved interpersonal interactions between clients and staff, and a less stigmatizing experience for clients. Because of this, the transformation process has held much appeal for charitable food agencies. To date, over 40 Minnesota food pantries have transformed, with more than 20 additional transformation requests in progress (47). Models like SuperShelf, which motivate agencies to make organizational changes, can potentially bolster the sustainability of nutrition interventions.

## 4. Food pantries as community hubs

In addition to destigmatizing the experience of going to a food pantry and designing the environment to maximize nutritious choices, food pantries present an opportunity to connect a vulnerable population with federal and local resources. For additional food, the U.S. federal government has 15 different food assistance programs, including the Supplemental Nutrition Assistance Program (SNAP), the Special Supplemental Nutrition Program for Women, Infants, and Children (WIC), and the National School Breakfast and Lunch Programs (SBP and NSLP) (48). Although there are people who go to food pantries in part because they don't qualify for federal assistance, there are also food pantry clients who may not have the knowledge or capacity to apply for these federal programs on their own and can be assisted in the food pantry setting. Food pantries could also connect clients with financial services, such as the Internal Revenue Service's Volunteer Income Tax Assistance program (49).

Partnerships between food pantries and the healthcare sector have also emerged around the country (50). Connections between these two systems allow communities to meet people where they are. In one direction, food pantries can serve as gateways into the healthcare system for the prevention and management of chronic diseases through screening, education, tailored meals, and clinical referrals (51, 52). Alternatively, clinicians in medical settings can screen patients for food insecurity and refer them to local food pantries (53). In clinics that serve high-risk populations, such as Federally Qualified Health Centers, food pantries may even be placed inside the clinic to maximize ease and access (54).

Meeting each individual's needs is key. Martin has developed an especially comprehensive and personalized framework called More Than Food (29), which was originally developed and tested in Hartford, CT (55). In these pantries, members are given access to a Coach for 9 months who uses motivational interviewing to help them identify specific goals and actions to take to reach those goals. Another example of a comprehensive approach is Community Food Centers Canada; this group describes itself as “an organization with the mission to build health, belonging and social justice in low-income communities across Canada through the power of food” (56).

## 5. Food pantries and civic engagement

The expanded vision for the charitable food system described thus far remains focused on the individual clients and their families and connecting them with the nutritious food and other resources they need to thrive. However, none of these programs or strategies will change the structure of our food system or the underlying societal issues that contribute to food insecurity. Those changes can only occur through policymaking and government regulation. The challenge is that many clients who use food pantries are focused on meeting their day-to-day needs and may not feel empowered to become civically engaged and try to change policies.

For most citizens, the most accessible avenue to exert influence is to vote. However, data on voting patterns suggests that low-income Americans are significantly less likely to vote than those with higher incomes and this has a significant impact on election outcomes (57). For this reason, there has been an increase in efforts for non-profits, including food banks and food pantries, to focus on voter engagement. In 2020, Feeding America collaborated with non-profit VOTE to use both digital and on-the-ground efforts to encourage clients to register to vote (58). Feeding America also provided grants to individual food banks, such as Philabundance, to work with their partner pantries in 2021 to provide information about the election and conduct voter registrations on-site (59). A 2022 report found that 37% of the food and nutrition non-profits surveyed reported conducting voter engagement at least occasionally (60). Future research is needed to identify how to build upon these efforts and provide non-partisan opportunities to educate clients about candidates and their positions.

## 6. Conclusion

The charitable food system has evolved significantly in the past decade. What began as a system of feeding low-income people food that would otherwise be wasted has evolved into a network that provides communities with resources to provide nutritious foods and access to additional resources. Moving beyond assisting individual families, the future power of food banking may be its access to people who are not yet civically engaged and empowering them to vote and get involved in shaping policies that will reduce the problem of food security for all.

## Author contributions

All authors listed have made a substantial, direct, and intellectual contribution to the work and approved it for publication.
